# Synchronous Wildfire Activity Rise and Mire Deforestation at the Triassic–Jurassic Boundary

**DOI:** 10.1371/journal.pone.0047236

**Published:** 2012-10-15

**Authors:** Henrik I. Petersen, Sofie Lindström

**Affiliations:** 1 Department of Reservoir Geology, Geological Survey of Denmark and Greenland (GEUS), Copenhagen, Denmark; 2 Department of Stratigraphy, Geological Survey of Denmark and Greenland (GEUS), Copenhagen, Denmark; New York State Museum, United States of America

## Abstract

The end-Triassic mass extinction event (∼201.4 million years ago) caused major faunal and floral turnovers in both the marine and terrestrial realms. The biotic changes have been attributed to extreme greenhouse warming across the Triassic–Jurassic (T–J) boundary caused by massive release of carbon dioxide and/or methane related to extensive volcanism in the Central Atlantic Magmatic Province (CAMP), resulting in a more humid climate with increased storminess and lightning activity. Lightning strikes are considered the primary source of wildfires, producing charcoal, microscopically recognized as inertinite macerals. The presence of polycyclic aromatic hydrocarbons (PAHs) of pyrolytic origin and allochthonous charcoal in siliciclastic T–J boundary strata has suggested widespread wildfire activity at the time. We have investigated largely autochthonous coal and coaly beds across the T–J boundary in Sweden and Denmark. These beds consist of predominantly organic material from the *in situ* vegetation in the mires, and as the coaly beds represent a substantial period of time they are excellent environmental archives. We document a remarkable increase in inertinite content in the coal and coaly beds across the T–J boundary. We show estimated burning temperatures derived from inertinite reflectance measurements coupled with palynological data and conclude that pre-boundary late Rhaetian mire wildfires included high-temperature crown fires, whereas latest Rhaetian–Sinemurian mire wildfires were more frequent but dominated by lower temperature surface fires. Our results suggest a major change in the mire ecosystems across the T–J boundary from forested, conifer dominated mires to mires with a predominantly herbaceous and shrubby vegetation. Contrary to the overall regional vegetation for which onset of recovery commenced in the early Hettangian, the sensitive mire ecosystem remained affected during the Hettangian and did not start to recover until around the Hettangian–Sinemurian boundary. Decreasing inertinite content through the Lower Jurassic suggests that fire activity gradually resumed to considerable lower levels.

## Introduction

**Figure 1:**
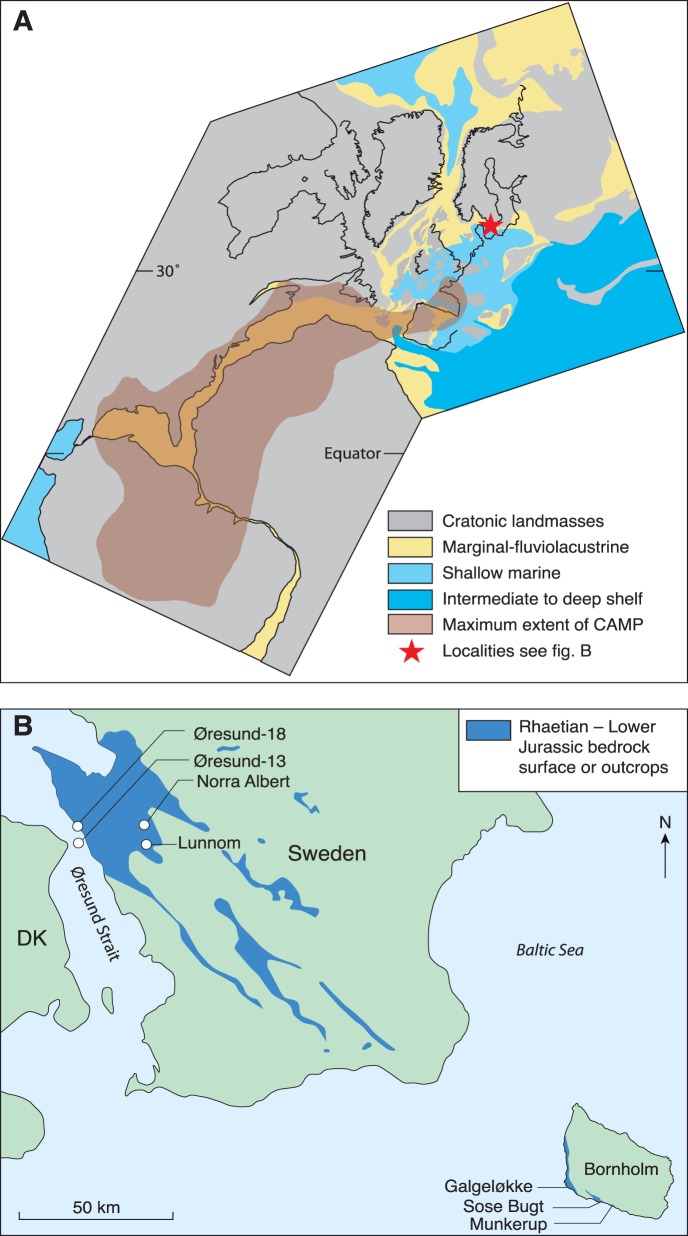
Palaeogeography at the Triassic–Jurassic boundary and location of the study area and investigated coal/coaly beds. A Palaeogeographic reconstruction of central Pangea showing the maximum extent of the Central Atlantic Magmatic Province (CAMP) (Modified after [7]). The location of the study area in the Danish Basin is marked by a red star. **B** Close up map of the study area, showing the localities/wells containing the investigated coal/coaly beds: Lunnom (Rhaetian, B-bed), Norra Albert (Rhaetian, B- and A-bed), Munkerup Member (lowermost Hettangian), Øresund-13 (lower Hettangian), Sose Bugt (uppermost Hettangian, D-bed; lowermost Sinemurian, B-bed), Øresund-18 (Sinemurian), and Galgeløkke (upper Sinemurian). DK  =  Denmark.

The major part of the Triassic was characterized by a dry, arid climate, albeit punctuated in mid-Triassic times by humid episodes with increased rainfall [1]. The end-Triassic, however, experienced a climatic change to a warm, humid greenhouse climate, as shown by peat (coal) deposition in the Rhaetian [2], kaolinite-rich mudstones at the Triassic/Jurassic (T–J) transition [3] and occurrence of soils typical for a humid tropical climate in lowermost Jurassic strata [4]. The opening of seaways from the Tethys into Pangea may have increased onshore precipitation in NW Europe through evaporation from the newly formed Rhaetian sea [2], [5]–[6]. The increased humidity may further have been intensified by the tremendous amounts of CO_2_ released to the atmosphere during the emplacement of the Central Atlantic Magmatic Province (CAMP), a large igneous province formed during the initial rifting of Pangea [7] (Fig. 1A). The temporal link between the CAMP volcanism and the end-Triassic mass extinction event [8]–[9] and major perturbations in the carbon cycle recorded in stable carbon isotope records globally are generally attributed to the effects of outgassing of ^12^C-enriched CO_2_ from this large igneous province [10] and/or from injection of methane to the atmosphere [11]–[13]. This is supported by palaeobotanical records, as stomatal density and the proportion of pores relative to epidermal cells on leaves are inversely related to the ambient CO_2_ concentration, and both numbers are reduced across the T–J boundary [14]. An estimated two- to fourfold increase in *p*CO_2_ recorded across the T–J boundary in Greenland, Sweden, and Northern Ireland [14]–[16] would potentially have caused extreme global warming, shallow water anoxia and a marine biocalcification crisis [17]. The end-Triassic mass extinction event affected both benthic and nektonic organisms with estimated losses of several marine families (23%) and genera (50%) on a global scale [17]–[18]. In the terrestrial realm regional to supraregional disappearances of vertebrate families (up to 42%) and plant species (up to 95%) have been recorded [14]–[15], [19]–[20]. Macrofossil plant [15] and palynological [7], [21] studies demonstrate a marked change from gymnosperm forests to fern-dominated vegetations across the T–J boundary. The proliferation of ferns is partly attributed to terrestrial acidification and pollution from emissions of SO_2_ and other toxic compounds from the CAMP volcanism [7]. Increased amounts of aerosols in the atmosphere due to the CAMP volcanism would have enhanced atmospheric water vapor content, which coupled with the inferred global warming is likely to have increased global storminess and lightning activity [22]. The presence of charcoal and pyrolytic PAH’s across the T–J boundary [7], [23]–[24], and a documented change in Greenland from a dominance of less flammable broad leaved plants to a more fire-prone vegetation dominated by narrow leaf morphologies [14], [23], supports a scenario of increased storminess and lightning activity. The aromatic carbon-rich chemical structure of charcoal make it resistant to degradation [25], and therefore charcoal can commonly be found as the allochthonous organic component in siliciclastic sediments. As elevated weathering rates and increased erosion is indicated by increased amounts of reworked palynomorphs in T–J boundary strata [26], there is a need to establish a fire record from more autochthonous strata.

Such records can be obtained from coal beds that consist of predominantly autochthonous organic material from the vegetation that grew in mires over a substantial period of time. “Mire” is here used as a general term for any freshwater wetland ecosystem in which organic matter (peat) accumulates (sensu [27]). This contrasts to continental siliciclastic beds that may represent relatively short depositional events. As peat mires are sensitive to environmental changes, coals are excellent environmental archives and their petrographic composition can record subtle changes in depositional conditions [28] and climate. Charcoal can be microscopically recognized in coals as macerals (semifusinite, fusinite, inertodetrinite, macrinite) of the inertinite group [29]–[31]. Therefore, the proportion of inertinite in coals provides an excellent record of the activity and nature of wildfires during deposition of the precursor peats.

The marginal uppermost Triassic (Rhaetian) and lowermost Jurassic (Hettangian–Sinemurian) succession of the Danish Basin preserves a unique suite of coaly beds in southern Sweden, in the Øresund strait between Denmark and Sweden, and on the Danish island of Bornholm in the Baltic Sea (Fig. 1B). The position of the T–J boundary within the Danish Basin succession is well constrained by a number of events. One of the most prominent markers that is widely recognized all over the basin (based on log-patterns, sedimentology and biostratigraphy) is the late Rhaetian maximum flooding event MFS7 of [5]. In the more proximal parts of the basin it is recognized as a marine incursion in an otherwise predominantly terrestrial environment and the two Rhaetian coal beds, B- and A-bed, where deposited during this maximum flooding [2]. The MSF7 can also be used for correlation outside the Danish Basin, e.g. in Germany where it appears to correspond roughly to the onset of the *Contorta-*Beds, while in St Audrie’s Bay, Great Britain, it is correlated with the middle Westbury Formation [2], [26].

Palynostratigraphic events, both marine and terrestrial ones, help to constrain the age of the coal beds/coaly beds within the Danish Basin succession. In combination with the high-resolution bulk organic C-isotope record of the Stenlille succession in the Danish Basin these palynostratigraphic events and changes in the biotic record has allowed correlation between the Danish Basin succession and the T–J boundary record at St. Audrie’s Bay in the United Kingdom [26].

Amongst the marine palynological markers the last common occurrence (LCO) of the dinoflagellate cyst *Rhaetogonyalax rhaetica* commonly coincides with the top of the marine black shale associated with MFS7 [2], [26]. The most prominent terrestrial palynological marker is the LCO of the typical Rhaetian taxon *Ricciisporites tuberculatus* which tends to more or less coincide with the T–J boundary [26]. The first occurrence (FO) of *Cerebropollenites thiergartii,* the accessory marker for the T–J boundary GSSP at Kuhjoch, Austria [32], further helps to pinpoint the position of the boundary within the Danish Basin.

Both the Triassic and Jurassic coaly beds contain considerable amounts of inertinite that allows measurement of inertinite reflectances and establishment of burning temperatures and types of wildfires across the T–J boundary [33]–[35]. Burning temperature relates to the type of fire (ground, surface or crown fire; see below), and experimental work has shown that inertinite reflectance increases with increasing burning temperature [29], [36]–[38]. The precursor freshwater peat mires were established on coastal plains facing an open sea to the west. Here we examine the inertinite content and composition, and measure inertinite reflectances of samples across the T–J boundary from coal beds in southern Sweden (Rhaetian Bjuv Member), coaly beds on the island of Bornholm (Hettangian–Sinemurian Munkerup, Sose Bugt and Galgeløkke Members), and coal beds in the Øresund strait (Hettangian–Sinemurian Helsingborg and Döshult Members) (Fig. 1B). The coal beds represent primarily autochthonous peat formation whereas the coaly beds have a stronger allochthonous component, but overall the organic compositions are assumed principally to record the depositional (climatic) conditions at the time of peat accumulation. The inertinite compositions of the coal and coaly beds are thus ideal to investigate fire activity across the T–J boundary. We supplement these data with a palynological analysis of 18 samples from 8 of the coal/coaly beds within the Rhaetian to Sinemurian interval that provides palaeoecological information on the mire ecosystems.

**Figure 2:**
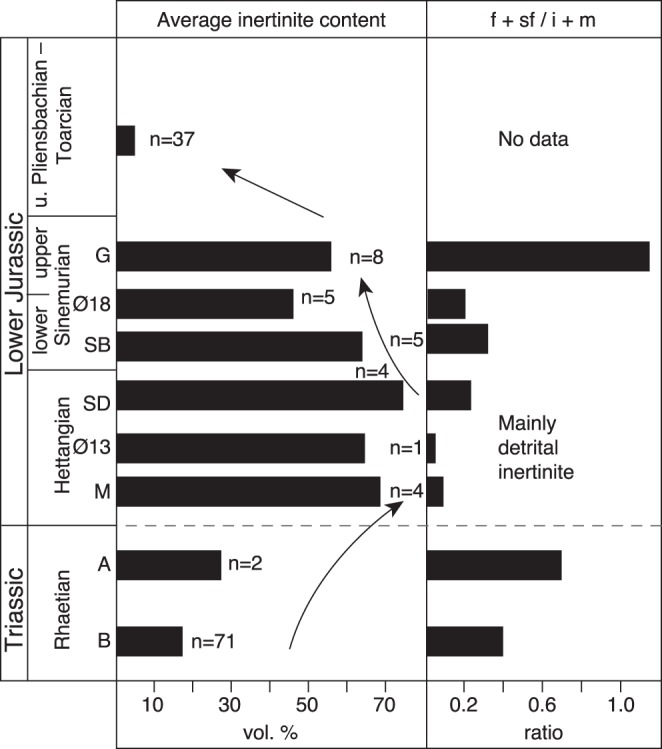
The average inertinite content of coal and coaly beds straddling the Triassic–Jurassic boundary . A significant increase in inertinite is recorded across the boundary with a maximum content in the Hettangian. Most of the inertinite in the Rhaetian A-bed and the Hettangian and lower Sinemurian beds is detrital (inertodetrinite). B: B-bed, Scania; A: A-bed, Scania; M: Munkerup, Bornholm; Ø13: Øresund-13 well; SD: Sose Bugt bed D, Bornholm; SB: Sose Bugt bed B, Bornholm; Ø18: Øresund-18 well; G: Galgeløkke, Bornholm.

## Materials and Methods

### Sample Material: Coal and Coaly Bed Descriptions

A comprehensive description of the coal and coaly beds used in this study is provided below. No specific permissions were required for the described field locations as they are public areas and are not protected in any way. An integrated comparison of the depositional environments and vegetation of the Early and Middle Jurassic mires on the island of Bornholm and in the Øresund strait between Denmark and Sweden can be found in [35]. The samples are channel samples and were collected from base to top of the studied coal and coaly beds in order to represent the entire bed.

#### Rhaetian

The coal samples were collected from the A-bed and B-bed of the Bjuv Member (Höganäs Formation) in Scania, southern Sweden. The lower B-bed and the upper A-bed define the Bjuv Member, and the top of the A-bed forms the lithostratigraphic boundary to the coarse and poorly sorted sandstones of the so-called Boserup beds [39], the the lowermost part of the uppermost Rhaetian to Hettangian Helsingborg Member (Höganäs Formation). The A and B coal beds were thus formed in the late Rhaetian very close to the T–J boundary. The sedimentary succession of the Höganäs Formation straddling the T–J boundary was deposited at the margin of the Danish Basin and consists of fluvial and deltaic sandstones, coals (A- and B-bed), and restricted marine to tidal heteroliths [2], [40]–[41]. A maximum marine transgression was recognized between coal beds B and A [2], and it has been correlated to the late Rhaetian maximum flooding event (MFS7) recognized in the Danish Basin [5].

**Table 1:** List of known or probable parent plant affinity of the identified spores and pollen, and preferred habit and environment.

Pollen	Known or probable parent plant affinity	Habit, preferred environment
*Alisporites* spp.	Seed fern, Corystospermales	upper canopy, mire
*Araucariacidites australis*	Conifer, Auracariaceae	upper canopy, well drained, coastal
*Cerebropollenites thiergartii*	Conifer	upper canopy, well drained
*Cerebropollenites macroverrucosus*	Conifer	upper canopy, well drained
*Chasmatosporites apertus*	Cycads/Ginkgos	upper canopy, mire
*Chasmatosporites elegans*	Cycads/Ginkgos	upper canopy, mire
*Chasmatosporites hians*	Cycads/Ginkgos	upper canopy, mire
*Clavatipollenites hughesii*	unknown gymnosperm	unknown habit
*Classopollis* spp.	Conifer, Cheirolepidiaceae	upper canopy, well drained, coastal
*Exesipollenites* spp.	Conifer, Cupressaceae/Taxodiaceae	?upper canopy, mire
*Eucommiidites* spp.	Erdtmanithecales	?Mid canopy, mire
*Granuloperculatipollis rudis*	Conifer, Cheirolepidiaceae	upper canopy, well drained
*Lunatisporites rhaeticus*	Conifer	upper canopy, well drained
*Monosulcites minimus*	Bennettitales	mid canopy, mire
*Monosulcites* spp. (Others)	Cycads/Ginkgos/Bennettitales/Peltaspermales (Seed ferns)	upper canopy, mire, drier patches
*Ovalipollis ovalis*	?Conifer	?well drained, unknown habit
*Perinopollenites elatoides*	Conifer, Cupressaceae/Taxodiaceae	upper canopy, mire, river banks
*Pinuspollenites minimus*	Conifer, Pinaceae	upper canopy, well drained
*Protohaploxypinus spp.*	Conifer	upper canopy, well drained
*Quadraeculina anellaeformis*	unknown gymnosperm	unknown habit
*Rhaetipollis germanicus*	unknown gymnosperm	unknown habit
*Ricciisporites tuberculatus*	unknown gymnosperm	?Mid canopy, ?mire
*Spheripollenites* spp.	Conifer	unknown
*Vitreisporites spp.*	Seed fern, Caytoniales	mid canopy, mire
**Spores**	**Known or probable parent plant affinity**	**Habit, preferred environment**
*Aratrisporites* spp.	Lycophyte	ground cover, mire, coastal
*Baculatisporites comaumensis*	Fern, Osmundaceae	ground cover, mire
*Baculatisporites opressus*	Fern, Osmundaceae	ground cover, mire
*Calamospora tener*	Equisetales	ground cover, mire, river banks
*Cibotiumspora juriensis*	Fern	ground cover, mire
*Cingulizonates rhaeticus*	Lycophyte	ground cover, mire
*Conbaculatisporites mesozoicus*	Fern	ground cover, mire
*Conbaculatisporites spinosus*	Fern	ground cover, mire
*Converrucosisporites* spp.	Fern	ground cover, mire
*Deltoidospora* spp.	Fern, Dipteridaceae, Dicksoniaceae	understory, mire, drier patches
*Densoisporites* spp.	Lycophyte, Pleuromeiaceae	ground cover, mire, coastal
*Densosporites* spp.	Lycophyte	ground cover, mire
*Gleicheniidites* spp.	Fern, Gleicheniaceae	ground cover, mire, drier patches
*Gordonispora fossulata*	Bryophyte	ground cover, mire
*Iraquispora laevigata*	Fern	ground cover, ?drier patches
*Laevigatosporites spp.*	Fern, Marattiales	ground cover, mire
*Limbosporites lundbladii*	Lycophyte	ground cover, mire
*Lycopodiacidites rugulatus*	Fern	ground cover, mire
*Marattisporites scabratus*	Fern, Marattiales	ground cover, mire
*Matonisporites* spp.	Fern, Matoniaceae	ground cover, mire
*Osmundacidites wellmanii*	Fern, Osmundaceae	ground cover, mire
*Punctatisporites* spp.	Fern, Osmundaceae	ground cover, mire
*Polycingulatisporites* spp.	Bryophyte	ground cover, mire
*Polypodiisporites polymicroforatus*	Fern, Schizaceae	ground cover, mire
*Polypodiisporites* spp.	Fern, Schizaceae, Polypodiaceae	ground cover, mire
*Retitriletes* spp.	Fern	ground cover, mire
*Skarbysporites crassexina*	Fern	ground cover, mire
*Stereisporites* spp.	Bryophyte	ground cover, mire
*Striatella seebergensis*	Fern, Polypodiaceae	ground cover, mire
*Thymospora* spp.	Fern, Marattiales	ground cover, mire
*Tigrisporites microrugulatus*	Fern	ground cover, mire
*Trachysporites* spp.	Fern	ground cover, mire
*Triancoraesporites ancorae*	Lycophyte	ground cover, mire
*Uvaesporites reissingerii*	Lycophyte	ground cover, mire
*Zebrasporites interscriptus*	Fern	ground cover, mire

**Table 2:** Coal/coaly bed relative palynomorph abundance data arranged after parent plant affinity.

		Fern allies			Ferns		Conifers				1Gi/Cy/Be		Unknown		Seed ferns		Unb^17^	Ma^18^
Locality	Sample	Br^2^	Eq^3^	Ly^4^	Gf^5^	Tf^6^	Ch^7^	Cu/Ta^8^	Pi^9^	Oc^10^	All^11^	Be^12^	Er^13^	*R* 14	All^15^	Ca^16^		
**Galgeløkke**	**2772**	3.0	8.0	0.3	2.0	38.0	1.0	17.0	1.0	3.0	2.3	0.7	14.0	0	2.7	0.3	3.0	3.7
**Øresund-18**	**6371**	0	1.0	0	3.0	30.0	0.3	24.0	11.0	1.0	2.0	0	0.7	0	13.7	0.3	9.0	4.0
**Øresund-18**	**6372**	0	0	0	0	40.0	4.0	22.0	4.0	0	0	4.0	0	0	9.0	0	13.0	4.0
**Øresund-18**	**6373**	0	2.0	2.0	3.0	56.0	0	4.0	4.0	0	4.0	0	0	0	4.0	0	13.0	8.0
**Sose Bugt B**	**6340**	3.0	8.0	0	4.0	32.0	1.0	19.0	5.0	1.0	5.0	2.0	1.0	0	3.0	0	2.0	14.0
**Sose Bugt B**	**6338**	11.0	3.0	1.0	4.0	41.0	2.0	11.0	3.0	0.3	1.0	0	0.3	0	5.0	0	16.0	1.3
**Sose Bugt B**	**6336**	7.0	1.0	1.0	4.0	48.0	0.3	11.0	4.0	1.0	3.0	0	1.0	0	2.0	0	9.0	7.7
**Sose Bugt D**	**6334**	3.0	3.0	1.0	7.0	48.0	0.7	6.0	9.0	1.0	2.0	0	2.0	0	5.0	0	6.0	6.3
**Sose Bugt D**	**6332**	8.0	4.0	1.0	5.0	33.0	1.0	4.0	13.0	3.0	4.0	1.0	1.0	0	6.0	1.0	9.0	6.0
**Øresund-13**	**247948**	0	0	0.7	8.0	54.0	0	0.7	14.0	1.0	0	3.0	0	0	11.0	0	5.0	2.7
**Munkerup**	**2765**	2.0	2.0	2.0	11.0	42.0	1.0	11.0	0.7	3.0	5.0	1.0	0	0	1.0	1.0	5.0	12.3
**Munkerup**	**2763**	0	2.0	1.0	9.0	56.0	0	6.0	1.0	0	3.0	2.0	0.7	0	1.0	0	4.0	14.4
**A-bed**	**18826**	1.0	0.3	1.0	28.0	33.0	2.0	2.0	0	2.0	0	14.0	0	1.0	2.0	10.0	2.0	1.7
**A-bed**	**18825**	1.0	0.7	2.0	46.0	12.0	1.0	4.0	0	1.0	2.0	8.0	0.3	4.0	2.0	9.0	3.0	4.0
**B-bed**	**18819**	0	0	4.0	28.0	5.0	2.0	23.0	0.3	3.0	0	8.0	0.7	1.0	2.0	19.0	3.0	1.0
**B-bed**	**18816**	0	0.5	0	13.0	0	1.0	58.0	0	0	4.0	4.0	0	0	0	11.0	3.0	5.5
**B-bed**	**18814**	0.3	0.3	3.0	27.0	6.0	4.0	18.0	0	2.0	4.0	16.0	0.3	3.0	1.0	5.0	6.0	4.0
**B-bed**	**18813**	0	0	2.0	15.0	7.0	4.0	38.0	0	3.0	6.0	10.0	0.5	1.0	1.0	6.0	2.0	4.5

1 Gingko/Cycads/Bennettitales.

2 Bryophytes.

3 Equisetopsids.

4 Lycophytes.

5 Ground ferns.

6 Tree ferns: *Deltoidospora* spp.

7 Cheirolepidaceae.

8 Cupressaceae/Taxodiaceae.

9 Pinaceae.

10 Other conifers.

11 All Ginkgo/Cycads/Bennettitales except Bennettitales^12^.

12 Bennettitales: *Monosulcites minimus.*

13 Erdtmanithecales, *Eucommiidites.*

14 *Ricciisporites tuberculatus.*

15 All seed ferns, except Caytoniales^16^.

16 Caytoniales: *Vitreisporites* spp.

17 Unidentified bisaccates.

18 Microalgae.

**Table 3:** Mire floral composition of the coal/coaly beds from the Danish Basin.

Locality	Sample	Ground cover (%)	Understory (%)	Mid canopy (%)	Upper canopy (%)	Sum
Galgeløkke	2772	14.6	41.6	19.0	24.8	100
Øresund-18	6371	5.3	39.6	4.0	51.1	100
Øresund-18	6372	0	53.3	5.3	41.3	100
Øresund-18	6373	9.3	74.7	5.3	10.7	100
Sose Bugt B	6340	19.2	41.0	10.3	29.5	100
Sose Bugt B	6338	24.5	52.8	1.7	21.0	100
Sose Bugt B	6336	16.5	60.8	5.1	17.7	100
Sose Bugt D	6334	17.9	61.5	5.1	15.4	100
Sose Bugt D	6332	25.4	46.5	9.8	18.3	100
Øresund-13	247948	11.1	68.9	3.8	16.2	100
Munkerup	2765	21.0	51.9	8.6	18.5	100
Munkerup	2763	14.9	69.4	7.0	8.7	100
A-bed	18826	32.2	35.0	26.5	6.4	100
A-bed	18825	54.0	13.0	25.4	7.6	100
B-bed	18819	34.2	5.3	30.6	29.9	100
B-bed	18816	14.9	0	21.0	64.1	100
B-bed	18814	35.7	7.0	39.9	17.4	100
B-bed	18813	19.0	7.8	24.0	49.2	100

A total of 73 coal samples were available from small quarries at Lunnom where the B-bed is exposed and at Norra Albert quarry (Billesholm) where the A- and B-beds are exposed. The B-bed is approximately 70–80 cm thick, and it is at Lunnom intersected by a thin clay layer containing the suessiacean dinoflagellate cyst *Lunnomidinium scaniense* [42]. The A-bed is only about 20 cm thick. Unpublished data [43] and data from new analyses of samples collected in Norra Albert quarry by the authors were available. The thicker B-bed consists of intercalated intervals of coal and coaly mudstone. Huminite dominates the maceral composition, whereas the liptinite content is low. The inertinite content (fossil charcoal) varies on average from 10–21 vol.% (mineral matter free, m.m.f.). The A-bed is richer in inertinite, reaching 27 vol.% (m.m.f.) on average.

**Figure 3:**
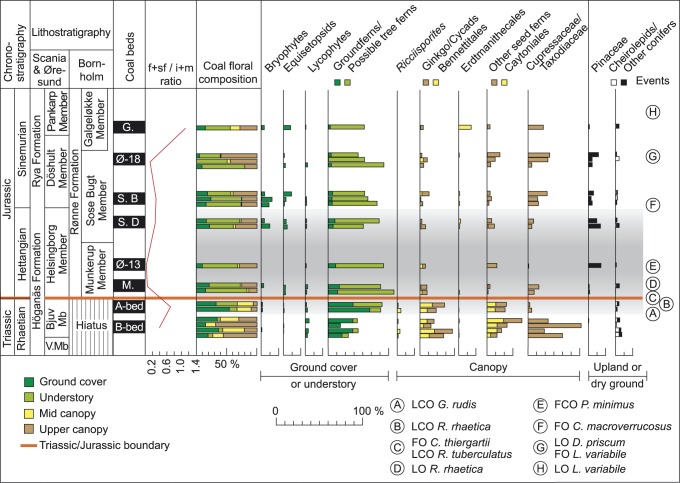
Mire floral composition from coal/coaly beds across the T–J boundary based on spores and pollen. Chrono- and lithostratigraphy is based on [2] and [5]. V.Mb  =  Vallåkra Member. The stratigraphic positions of the coal/coal beds are based on previous studies [2], [33]–[35] and on the palynological data in this paper. The detailed f+sf/i+m ratio (red curve) demonstrates a dominance of detrital inertinite in the Hettangian coal/coaly beds. The coal floral composition is calculated from spore/pollen abundances of probable mire taxa only, while the total abundances of the various plant groups are based on all spores and pollen, including those regarded to have been produced by upland or dry ground plants. The Ginkgos/Cycads/Bennetittales category probably also includes pollen produced by peltasperms (seed ferns). A to E represent palynostratigraphic events, both marine and terrestrial, where FO  =  First occurrence, FCO  =  First common occurrence, LO  =  Last occurrence, and LCO  =  Last common occurrence. A: LCO of *Granuloperculatipollis rudis*, B: LCOs of *Rhaetogonyaulax rhaetica* and *Ricciisporites tuberculatus*, C: FO of *Cerebropollenites thiergartii*, the accessory marker for the T–J boundary, D: LO *R. rhaetica*, E: FCO of *Pinuspollenites minimus*, F: FO of *Cerebropollenites macroverrucosus*, G: LO of *Dapcodinium priscum* and FO of *Liasidium variabile*, and H: LO of *Liasidium variabile*. Thick red line marks the T–J boundary.

#### Hettangian

A total of nine coaly samples were derived from the Munkerup and Sose Bugt Members (Rønne Formation [44]) on the island of Bornholm in the Baltic Sea and from the Øresund-13 well located in the Øresund strait between Denmark and Sweden.

The sediments of the Munkerup Member were deposited in a lake-dominated wetland [44]–[45]. The setting was mainly freshwater, but some pyrite in the coaly beds could suggest limited influence from saline water [33]. The Munkerup beds are between 12 cm and 30 cm thick and represent mixed allochthonous and autochthonous coaly deposits as evidenced by a high proportion of detrital macerals (mainly inertodetrinite), a high clay content, and the presence of huminite and root horizons. The average inertinite content is about 71 vol.% (m.m.f.). The presence of huminite indicates restricted oxygen availability in the precursor mires, and coupled with the dominant clay fraction and the occasionally faint lamination in the coaly beds, open mires with relative stagnant water are envisaged. The inertinite is considered to be both allochthonous and autochthonous, and it is considered most likely that the allochthonous inertinite was formed nearby the mires and had a short transport distance before deposition.

**Figure 4:**
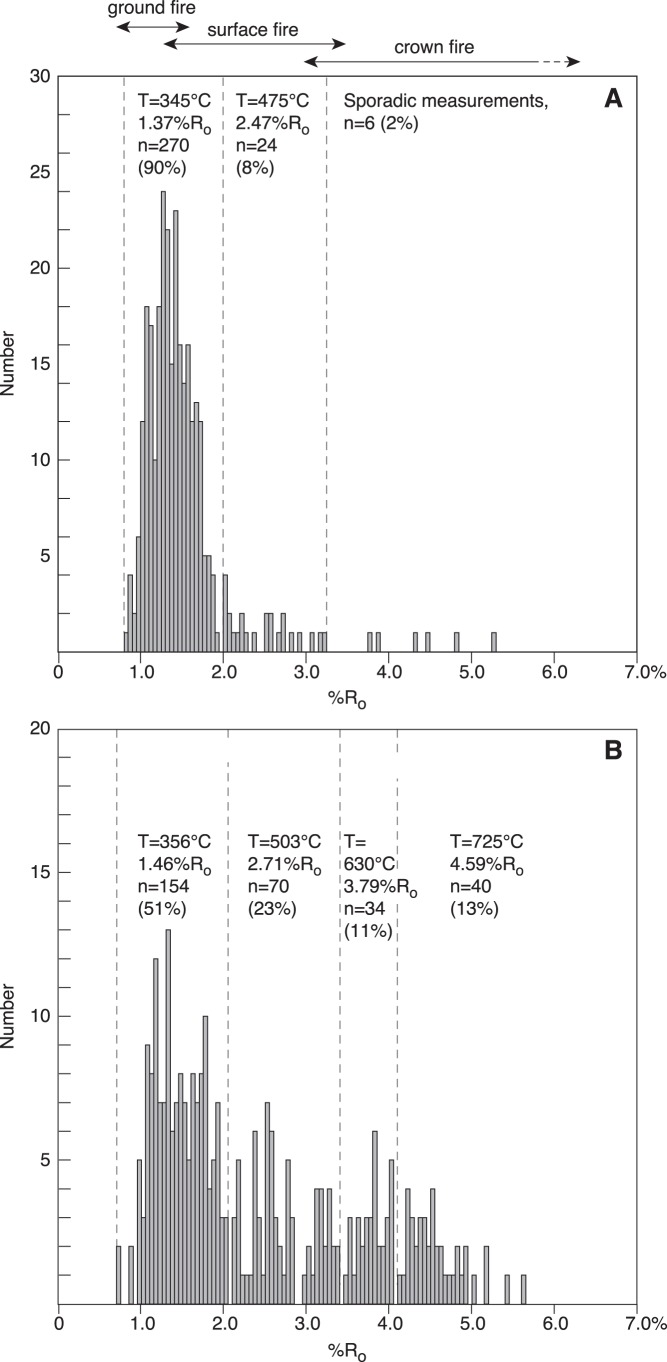
Inertinite reflectance histograms showing calculated burning temperatures of selected reflectance intervals and the possible fire types. A Sample from the Hettangian Munkerup Member (section 2, bed 1, sample 2763/M-3-1.2). Most inertinite has low reflectance suggesting it was primarily derived from surface fire. B Sample from the B-bed in the Rhaetian Bjuv Member (Norra Albert quarry, sample 18812). Almost 25% of the inertinite is high-reflecting suggesting it was derived from high temperatures reached in crown fires.

**Figure 5:**
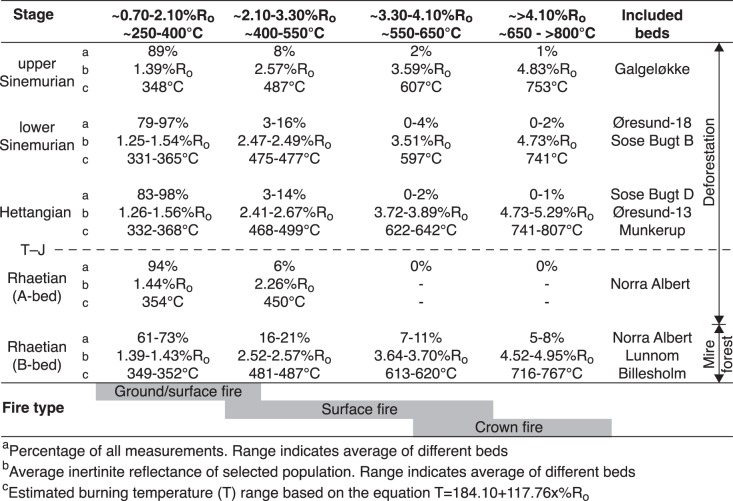
Inertinite reflectance populations and assumed burning temperatures. High-reflecting inertinite corresponding to temperatures >600°C that can be obtained in crown fires of stands of conifers is particular abundant in the Rhaetian B-bed.

**Figure 6:**
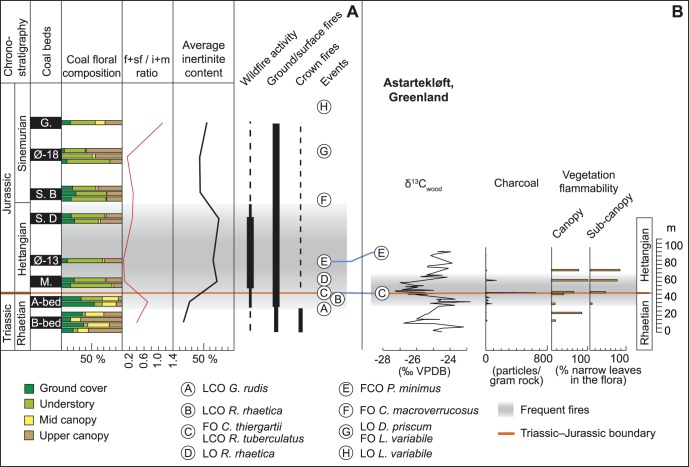
Comparison of the Danish Basin wildfire record with the charcoal record from Greenland. A Position of coal/coaly beds, f+sf/i+m ratio, Average inertinite content, interpreted wildfire record and events for the Danish Basin showing shift from common crown fires to dominance of surface fires and continued high wildfire activity in mires during the entire Hettangian. **B** Wood Carbon-isotope record [10], charcoal record and vegetation flammability record indicating increased fire activity and vegetation adaptations across the T–J-boundary [23] for the Astartekløft T–J-boundary succession in Greenland.

The 15 cm thick coaly bed D of the Sose Bugt Member is regarded to be of late? Hettangian age [46]. Macroscopically it appears hard and structureless. The coaly bed is clay-rich and was deposited in an open freshwater mire. The coaly bed contains some huminite but the organic matter is dominated by low-reflecting inertinite (averaging 75 vol.%, m.m.f.), mainly occurring as inertodetrinite.

**Figure 7:**
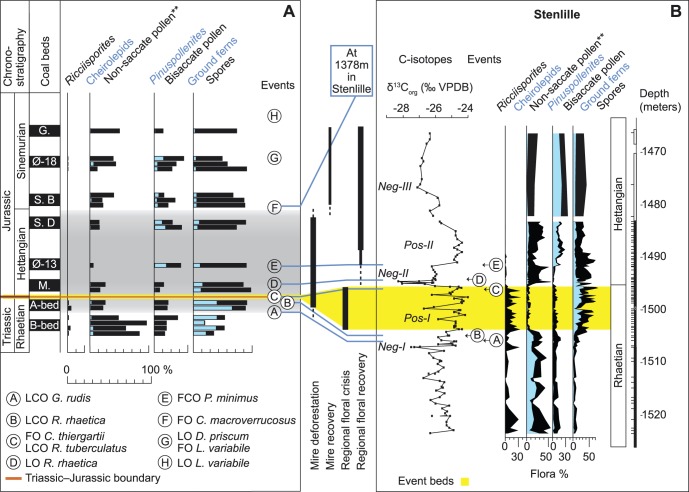
Correlation of the regional palynological signal. Correlation of **A** the overall palynological record from the coal/coaly beds of the Danish Basin with that of **B** the Stenlille record [26], showing onset of mire deforestation probably commenced prior to regional deforestation (yellow field), and delayed mire ecosystem recovery compared to the overall regional vegetation.

The coaly bed in the Øresund-13 well was formed in an oxygen-deficient freshwater environment as a result of infilling of a restricted bay or lagoon and decreasing water depth [34], [47]. The bed is characterized by a high proportion of detrital macerals, mainly inertodetrinite, sorting of the organic matter and microlamination, which has been interpreted to indicate an open mire with a sparse *in situ* vegetation as shown by rootlets [34]. Part of the allochthonous inertinite was suggested to have been derived from redeposition of peat from adjacent areas.

#### Lower sinemurian

A total of 18 coal and coaly samples were available from the Sose Bugt Member (Rønne Formation [44]) on the island of Bornholm and from the Øresund-18 well in the Øresund strait.

The coal bed B from the Sose Bugt Member occur in a paralic succession containing lacustrine, fluvial and restricted marine deposits [46]. Overall the sedimentary section describes a backstepping lacustrine to estuarine succession. Macroscopically the 30 cm thick coaly bed B appears clayey, hard and structureless [33]. The mineral content is high and the bed was deposited in an open mire with a sparse vegetation. The coaly bed contains some huminite, part of which may represent root tissues. The content of low-reflecting inertinite amounts to about 66 vol.% (m.m.f.), the main part composed of inertodetrinite.

Two coal beds (bed 1 and 2) occur in the Øresund-18 well on top of lake sediments that describe the gradual infill of freshwater lakes [34]. Coal bed 1 has been included in this study. The coal bed is 25 cm thick and comprise black, compact coal. The coal bed is generally rich in low-reflecting inertinite, averaging 47 vol.% (m.m.f.), that largely consists of poorly sorted and angular inertodetrinite. The inertinite is considered to be formed *in situ* in relatively dense vegetated mires. Autochthonous peat formation is indicated by the presence of huminite (average 42 vol.%, m.m.f.) and the presence of rootlets below the coal beds. Coal bed 1 may have been slightly domed.

#### Upper sinemurian

Samples were derived from a coal bed in the Galgeløkke Member (Rønne Formation [44]) on the island of Bornholm. The freshwater coal bed overlies tidal flat sediments and thus marks a significant change in the depositional environment [35]. The coal bed may have a faint lamination and is generally dull, but sometimes lustrous with visible fusinite particles (charcoal) [33]. The coal bed has been interpreted to represent an autochthonous raised peat deposit, possibly subjected to a fluctuating watertable. It contains a low content of mineral matter and a high content of inertinite (about 55 vol.%, m.m.f.) with varying reflectance. The inertinite is composed of both fusinite and inertodetrinite.

### Inertinite Reflectance Measurements

Pellets of the coal and coaly samples suited for reflected light microscopy were prepared. The samples were lightly crushed and sieved between 63 µm and 1 mm in order to get the analysis fraction. This fraction was embedded in epoxy, and the epoxy pellets were ground and polished to obtain a smooth surface. Inertinite reflectance measurements (random, oil immersion) were conducted using a Leitz Orthoplan reflected light microscope equipped with a 32× objective and the Diskus Fossil vitrinite reflectance system (Hilgers Technisches Buero, Germany). The reflectance measurements were taken at 546 nm (monochromatic light). Before measurement the microscope was calibrated against a YAG 0.903%R_o_ standard and an optical black (zero) standard. The Diskus Fossil system is software controlled and uses no photomultiplier which makes it very robust and linear to high reflectance values. It was therefore considered sufficient in this context only to use the YAG 0.903%R_o_ standard. Generally 7×7 pixels (1.4 µm^2^) were used as measure spot size, but the spot size was reduced or enlarged if considered appropriate. This had no effect on the measured reflectance values. A total of 297 to 300 measurements were taken per sample (33 samples in total). Average inertinite reflectance values corresponding to different burning temperatures were calculated from selected reflectance populations from the total reflectance histograms. The definition of the intervals was guided by established inertinite reflectance-temperature-fire type relationships [36]–[38], [48].

### Inertinite Contents

The inertinite contents of Rhaetian, Hettangian and Sinemurian coal and coaly beds were calculated from maceral analyses of 100 samples. In addition, the average inertinite content of 37 upper Plienbachian–Toarcian coal samples are shown in Figure 2. The maceral compositions have been determined by incident light microscopy and oil immersion. The organic matter was analysed in reflected white light and fluorescence-inducing blue light. A total of 500 macerals and minerals have been point-counted in each sample using an automatic point counter (Swift) that generates a uniformly spaced grid of points. The point-count analysis yields the proportion of each maceral and maceral group in vol.%. Analysis and maceral identifications followed international standards [30], [49]–[50]. Previously obtained data and new analyses have been included. New Rhaetian data were derived from maceral analysis of samples from Norra Albert quarry in southern Sweden, whereas other Rhaetian data were calculated from a public available Thesis [43]. Published data were used to calculate the inertinite content of Hettangian and Sinemurian coal beds in the Øresund area [34]. The inertinite content of the Hettangian, Sinemurian and upper Plienbachian–Toarcian samples from the island of Bornholm were calculated from mainly unpublished data produced by the first author (H.I. Petersen).

### Palynological and Palaeoecological Analysis

For palynology, ca 20 g of bulk rock from twenty-four of the analyzed coal and coaly samples from the B-bed and A-bed, and from the Munkerup, Øresund-13, Øresund-18, Sose Bugt and Galgeløkke beds were selected for analysis. The coal/coaly samples were treated in alternating steps with hydrochloric (38%) and hydrofluoric acid (40%) to remove carbonate and silicate mineral matter. After washing to neutrality, residues were sieved with 11 µm mesh-size sieves and mounted on 2–4 strew slides. Six of the coal/coaly samples were either barren of palynomorphs or contained too few to be statistically significant, and they were therefore excluded from the quantitative analysis. Up to 300 palynomorphs were counted in one slide per sample with a compound microscope at 650× magnification. Abundance data were calculated as percentages of total palynomorph assemblage. The spores and pollen were divided into taxa groups reflecting their known or probable parent plant affinity, based primarily on documented *in situ* occurrences of spores and pollen [51] (Tables 1 and 2). The various parent plant affinity groups were then subdivided after their probable preferred habitat: ground cover, understory, mid canopy and upper canopy (Tables 1, 2, 3), based on palaeobotanical analyses from Greenland [15] and Scania [52]. The ground cover category includes all spores within bryophytes, equisetopsids, lycopsids and ferns, except *Deltoidospora*. Spores of the latter form genus can be attributed to several different fern families including the Dipteridaceae, Matoniaceae, Dicksoniaceae, and the Cyathaceae. However, macroplant *in situ* occurrences in Triassic–Jurassic strata within NW Europe only include the Dipteridaceae (T–J boundary strata from Germany and Greenland [51]) and the Dicksoniaceae (Triassic strata from Sweden, and Jurassic strata from England and Sweden [51]). Extant dipterid ferns are ground ferns, while extant Dicksoniaceae are tree ferns [53]. Because *Deltoidospora* exhibits a remarkable increase in abundance across the T–J boundary it is placed in a category of its own; tree ferns. However, it should be noted that prior to the Upper Jurassic there is no palaeobotanical evidence that the Dicksoniaceae had trunks [53]. The mid canopy category contains monosulcate pollen grains assigned to *Monosulcites minimus* and the bisaccate pollen *Vitreisporites,* that are of bennettitalean and caytonialean affinity [51], respectively. The enigmatic heavily sculptured monosulcate pollen *Ricciisporites tuberculatus* found in permanent tetrads is also included within the mid canopy category, however, neither its parent plant affinity nor its preferred habitat is known to date [54]. Also included in the mid canopy category is pollen assigned to *Eucommiidites* which belongs to the Erdmanithecales. The remaining monosulcate pollengrains are believed to be of Ginkgo/Cycad/Bennettitalean affinity, although some may have been produced by peltasperms [51], and are included in the upper canopy category because ginkgos appear to have been a major part of the upper canopy in Greenland [15]. Other seed ferns mainly include bisaccate pollen assigned to *Alisporites* and these are included in the upper canopy category. Pollen assigned to *Perinopollenites elatoides* are of taxodiacean/cupressacean affinity, having been found *in situ* in various *Elatides* and *Masculostrobus* cones from the Jurassic of England and Scotland [51], [53]. These pollen are believed to have been produced by upper canopy trees like *Stachyotaxus* that thrived in wet environments [15]. In contrast, conifer pollen assigned to *Pinuspollenites* and the circumpolloid group, e.g. *Classopollis*, that have affinities with the Pinaceae and the Cheirolepidaceae, respectively, are not considered to have been mire floral elements, and are thus not included in the quantitative assessment of the composition of the mire flora (Table 3). Both the Pinaceae and the Cheirolepidaceae are regarded to have preferred more well-drained conditions, on drier ground or in upland areas.

## Results and Discussion

### Changes in Inertinite Content Across the T–J Boundary

The inertinite content in the coal/coaly beds shows a remarkable increase across the T–J boundary (Fig. 2). The late Rhaetian B-bed contains about 17 vol.% (m.m.f.) inertinite on average with two thirds of the samples containing less than 20 vol.% (m.m.f.) inertinite. The overlying Rhaetian A-bed shows an increase in inertinite to about 27 vol.% (m.m.f.), and a substantial increase in inertinite is observed in the Hettangian coaly beds where the average content of inertinite ranges from 66–75 vol.% (m.m.f.) with recorded maximum values in individual samples of 82 vol.% (m.m.f.). A slight decrease in average inertinite content is observed in the lower and upper Sinemurian beds (Fig. 2). For comparison the average inertinite content of 37 samples from upper Pliensbachian–Toarcian (upper Lower Jurassic) coal beds on Bornholm have been included and these show a dramatic decrease to only 4 vol.% (m.m.f.) [55] (Fig. 2). Several of the coal samples are lean in inertinite. The coaly beds thus reveal a significant increase in inertinite, i.e. fossil charcoal, across the T–J boundary. The inertinite consists of structured fusinite and semifusinite macerals showing preserved cell lumens, and detrital inertodetrinite and macrinite macerals with an overwhelming dominance of inertodetrinite. The amount of the individual inertinite macerals in the coaly beds cannot directly be compared because of the varying total amounts of inertinite in the beds, however, the ratio between structured fusinite+semifusinite and detrital inertodetrinite+macrinite (hereafter f+sf/i+m ratio) provides information about the relative proportions of these inertinite macerals. The f+sf/i+m ratio of the Rhaetian B-bed is 0.40, the Rhaetian A-bed ratio is 0.69, the Hettangian ratios range from only 0.04–0.24, the lower Sinemurian ratios range from 0.23–0.37, and the upper Sinemurian ratio is 1.16 indicating a pronounced dominance of detrital inertinite in the Hettangian coaly beds followed by a minor increase in structured inertinite in the lower Sinemurian (Fig. 2). The ratio of the B-bed may be lower than expected because the potentially structured inertinite formed by burning of trees at high burning temperatures (see below) may have been splintered into detrital inertinite. The A-bed ratio is an average of a relatively inertinite-poor (∼17 vol.%, m.m.f.) sample containing largely equal amounts of structured and detrital inertinite and an inertinite-rich (∼37 vol.%, m.m.f.) sample strongly dominated by detrital inertinite. The dominance of inertodetrinite may tentatively be interpreted to reflect charring of thin-walled less lignified wood as a result of a change to smaller plants (herbaceous and shrubby vegetation) across the T–J boundary. The floral change is confirmed by the fern-spore dominance in the Hettangian beds of the Munkerup Member on Bornholm and the Øresund-13 well in the Øresund strait (Fig. 3). The high abundance of fern spores and the high inertodetrinite content in the upper Rhaetian A-bed suggest that the mire-forest deterioration began in the latest Rhaetian. This finding is in agreement with results from Denmark, Sweden and Germany that showed replacement of arborescent vegetation with a flora dominated by ferns and fern allies during the latest Rhaetian [7], [26]. The relative higher content of fusinite and semifusinite in the Rhaetian B-bed would similarly agree with a higher proportion of trees in these precursor mires (Fig. 3). The Hettangian and lower Sinemurian coaly beds from Bornholm are mineral-rich (very fine-grained) and part of the inertinite is probably allochthonous or redistributed within the mires, but the fine-grained clayey mineral matter points to very slow-flowing water with little capacity to transport and separate charcoal particles. The hydrodynamic behavior of charcoal varies with size as larger particles take longer to saturate with water, and further charcoal formed at high burning temperatures is likely to settle faster because of extensive cracking and thus less buoyancy [56]–[57]. The indication of weak waterflow in the Hettangian to early Sinemurian mires and the surprisingly low measured inertinite reflectances (see below), indicating high buoyancy of the charcoal, suggest that the striking absence or low abundance of fusinite and semifusinite in the coaly beds is the result of non-formation of these macerals.

### Inertinite Reflectances: Implications for Fire Types and Vegetation

Wildfires have probably occurred since the latest Silurian when the land surface was colonized by plants and the atmospheric oxygen content had reached a level that could sustain fire [58]. Wildfires can be grouped into [29] (1) ground fires that burn organic material below the litter, (2) surface fires that burn litter and herbaceous and shrubby plants, and (3) crown fires that burn the canopy of trees and larger shrubs. The fire types are characterized by different burning temperatures with ground fires producing temperatures of ∼300°C albeit flames in many fires generally produce temperatures around 600°C; intense heat with temperatures of 800°C or higher can be reached in crown fires in for example stands of conifers [38], [48]. These temperatures reflect wildfires in modern ecosystems, where carring occurred under the prevailing atmospheric composition of ∼21% oxygen. Other oxygen levels may have impacted burning temperatures of wildfires. Further at a given temperature reflectance increases rapidly at first before leveling off over time. This suggests that the measured inertinite values can only be taken to equate to minimum burning temperatures. The reflectance of inertinite (charcoal) is related to burning temperature as documented by several experimental charring studies that showed increasing reflectance with increasing temperature [29], [31], [36]–[37], [48], [59]. The reflectance increase is caused by the carbon-rich aromatized structure formed by the charring process. Lignified wood has been suggested to yield higher reflectances than cellulose-rich tissues because of its inherited higher initial aromaticity [29]. This may suggest that an arborescent vegetation with much lignified wood has the potential to produce higher quantities of high-reflecting inertinite than herbaceous floras containing less lignified wood. The correlation between the inertinite reflectance distribution (see below) and the composition of the burned vegetation based on botanical affinities of palynomorphs is to some extent illustrated in Fig. 3. A very robust correlation between the petrology and the palynology of the coals cannot be expected because of the inherited uncertainty in using palynomorphs to reconstruct *in situ* vegetations [60]–[61]. Further, despite the studied coaly beds represent overall similar mire environments it is likely that subenvironments existed within the mires and in-seam variations reflecting mire development are also evident. Albeit the correlation between inertinite reflectance and burning temperature is not completely linear, the correlation has been described by the linear regression equation T = 184.10+117.76x%R_o_ (r^2^ = 0.91), where T is the burning temperature and %R_o_ is the measured inertinite reflectance [37].

The measured inertinite reflectance histograms of the Rhaetian to Sinemurian samples have been used to derive burning temperatures, fire types, and to suggest the possible predominant vegetation in the mires subjected to wildfire (Fig. 4). The reflectance distributions of the Rhaetian B-bed reveal several reflectance populations, but they are in particular characterized by two pronounced populations of high-reflecting inertinite with average reflectances ranging from 3.64–3.70%R_o_ and 4.52–4.95%R_o_ (Fig. 5). Measured maximum reflectance values exceed 5%R_o_. Using the above equation this high-reflecting inertinite can be estimated to have been formed at temperatures from ∼613–620°C and ∼716–767°C, respectively. The highest reflectance values indicate burning temperatures >800°C. These temperatures suggest crown fires, probably in conifers that can produce intense heat (Figs. 5, 6A). In Yellowstone National Park reflectances of up to 6%R_o_ were attributed to crown fires in stands of conifers [38]. In the fossil record high inertinite reflectances above 6%R_o_ have been recorded in Upper Jurassic coaly mudstones from northeast Greenland where average inertinite reflectance values from 4.65–5.16%R_o_ were calculated from well-defined populations in the high-reflectance range [62]. The presence of lower reflecting inertinite in the B-bed further indicates that surface fires and maybe also ground fires occurred in the mires (Fig. 5), suggesting that the precursor mires supported both stands of conifers and an understory of shrubs and herbs. Surface fires are most likely as ground fires are typically smouldering fires that consume charcoal [63]. Our palynological data from the B-bed corroborates this revealing a dominance of upper canopy tree pollen from taxodiaceous/cupressacean conifers (mean 34%) with accessory mid canopy bennettitalean (mean 9%) and caytonialean pollen (mean 11%) (Fig. 3). Common ground fern spores (mean 21%), primarily of marattialean affinity, represent ground cover plants. In contrast, the high-reflecting inertinite is not present in the overlying A-bed, which agrees with a dominance of herbaceous fern spores (mean 60%) in the bed, and a further remarkable increase in spores that may represent understory tree ferns (Dicksoniaceae/Cyathaceae; mean 22%). The inertinite reflectance distributions of the Hettangian coaly beds reveal a significant change in reflectance distribution (Figs. 4, 5). Inertinite reflectance values above approximately 3%R_o_ are virtually absent as only up to 2% of all measured particles are high-reflecting. Low-reflecting inertinite populations with average values ranging from 1.26–1.56%R_o_ accounts for 83–98% of the measurements (Fig. 5). This corresponds to burning temperatures of ∼332–368°C which suggests a predominance of surface fires in the Hettangian mires (Fig. 6A). The abundance of inertinite and the indication of dominantly low-temperature fires suggest high fire activity and charring of principally shrubby and herbaceous vegetations, probably because arborescent plants composed a minor part of the mire floras as suggested by the palynological data. The precursor mire of the lowermost Hettangian Munkerup coaly bed appears to have been a relatively sparsely forested mire, where upper canopy trees are few and mid canopy trees almost absent, as indicated by markedly decreased amounts of cupressacean/taxodiacean pollen (mean 8%) and rare pollen indicative of Bennetittales (mean 1.5%) and Caytoniales (0.5%) (Fig. 3). Instead the mire was dominated by probable tree ferns (mean 49%) and common ground ferns (mean 10%). In the lower Hettangian coaly bed in the Øresund-13 well the palynological pattern is similar with a dominance of fern spores, very few mid canopy pollen and in addition cupressacean/taxodiacean pollen are virtually absent (Fig. 3). In addition, the inertinite reflectance distribution is dominated by a major population that accounts for 83% of all measurements, yielding an average reflectance of 1.56%R_o_ indicating a predominance of surface fires (Fig. 5, 6A). This is in accordance with the registered potential loss of mid canopy trees observed in the macroplant record on Greenland [15], but also indicates a dramatic decrease in upper canopy trees within the mire with a marked relative increase in ground ferns and probable tree ferns. Ferns are commonly pioneering plants in disturbed ecosystems, including post-wildfires, as seen in the recent Okefenokee swamp in U.S.A. and in Middle Jurassic coal beds on Bornholm [64]. The charcoal record from Greenland shows a remarkable increase across the T–J-boundary with a distinct peak just above the boundary (as marked by the FO of *Cerebropollenites thiergartii*), and this is supplemented by a shift towards more fire prone vegetation as canopy and sub-canopy plants with narrow and dissected leaves become more common [23](Fig. 6B). The amount of charcoal recovered in the Greenland succession decreases during the early Hettangian, whereas the fire prone vegetation remains dominant (Fig. 6B). In contrast the wildfire record from the coal/coaly beds of the Danish Basin indicate continued high fire activity throughout the Hettangian (Fig. 6). The proportion of inertinite first decreases slightly in the Sinemurian coaly beds and in the upper Plienbachian–Toarcian coal beds the inertinite content is inferior (Fig. 2, 6). This indicates a decrease in wildfire activity through the remaining part of the Early Jurassic following Hettangian time with a dramatic decrease following Sinemurian time. The palynology shows an increase in upper canopy cupressacean/taxodiacean conifers in the lower Sinemurian and a possible return of mid canopy plants in the upper Sinemurian, indicating that by that time the mire ecosystem had become more densely structured.

The overall palynofloral composition of the Rhaetian to Sinemurian coals in part mirrors the palynological record of the marine sedimentary succession in the Stenlille wells [26], Denmark (Fig. 7). In the Rhaetian the last common occurrences (LCOs) of *Granuloperculatipollis rudis* and the dinoflagellate cyst *Rhaetogonyaulax rhaetica* in the Rhaetian coal beds or in the sediments associated with these coals confirm the correlation with the black shales and the MFS7 in the Stenlille succession [2], [5], [26]. The most apparent difference between the Rhaetian coal bed record and that of Stenlille is the higher frequencies of small monocolpates (*Monosulcites minimus*, Bennettitales) and bisaccates (*Vitreisporites*, Caytoniales) in the coal beds compared to in the marine Stenlille succession. The herein registered decrease in canopy trees within the Rhaetian A-bed indicate that the recorded floral deforestation commenced earlier in the more sensitive mire environment compared to the regional floral record (Fig. 7). The presence of *Cerebropollenites thiergartii*, the accessory marker for the T–J boundary [21], [65], as well as the last occurrence (LO) of *Rhaetogonyalax rhaetica* in the Munkerup coaly bed show that the precursor mire existed in the earliest Hettangian. Correlation with the Stenlille succession [26] indicates that the Munkerup coaly bed was deposited after the event beds of the Stenlille succession and just before or during the negative carbon isotope excursion Neg-II (Fig. 7). The first common occurrence of *Pinuspollenites minimus* in the coaly bed in the Øresund-13 well indicates an early to mid Hettangian age for its precursor mire. The increased level of *Pinuspollenites minimus* in the coaly bed mirrors the increase of that pollen in the Stenlille succession (Fig. 7). The extremily low amount of the Cupressacean/Taxodiacean pollen *Perinopollenites elatoides* in the Øresund-13 well possibly mirrors a decrease in that taxon in the mid Hettangian of the Stenlille record. The parent plant of *P. elatoides* is believed to have preferred wet lowland environments, including mires. The Sose Bugt D coaly bed shows a similar pattern with low amounts of *Perinopollenites.* The D-bed is stratigraphically constrained to the latest Hettangian by the presence of *Cerebropollenites macroverrucosus*, a marker for the Hettangian–Sinemurian boundary [66], in the succeeding Sose Bugt coal bed B. The first occurrence (FO) of *Cerebropollenites macroverrucosus* in the Sose Bugt B-bed confirms an early Sinemurian age for this coaly bed. It can be correlated with the FO of *C. macroverrucosus* in the Stenlille succession [26]. The continued presence of the dinoflagellate cyst *Dapcodinium priscum* in sediments associated with this coaly bed and that from Øresund-18 indicates an early Sinemurian age for these beds [67]. The presence of the dinoflagellate cyst *Liasidium variabile* in succeeding strata in the Øresund-18 well and in sediments associated with the Galgeløkke coal bed confirms a late Sinemurian age for this bed [68].

The studied coal and coaly beds provide a more autochthonous terrestrial palynological record representing the mire habitat in particular than the combined marine/terrestrial palynological succession in the Stenlille wells, which show a terrestrial ecosystem deterioration during the late Rhaetian. However, while the Stenlille palynological record reflects all terrestrial habitats in the area and shows onset of terrestrial ecosystem recovery in the early Hettangian [26], the coal and coaly beds palynology primarily represent the mire habitat, which clearly remained affected throughout the Hettangian.

## Conclusion

### Causes for Increased Fire Activity Across the T–J Boundary

Our results show a significant increase in fire activity in mires across the T–J boundary with a maximum fire frequency in the Hettangian. This was associated with a change from more forested mires in the late Rhaetian to more fern- and shrub-dominated mire floras in the latest Rhaetian, Hettangian and early Sinemurian as indicated both by calculated burning temperatures and palynology. Increased fire activity and deforestation thus appeared to start in the latest Rhaetian below the T–J boundary. These changes are in agreement with other studies that have shown deforestation [7], [15], [26], [69] and charcoal abundance in the latest Rhaetian close to the T–J boundary [16], [23], [24]. High concentrations of PAH’s in Hettangian strata from Poland [24] suggest the most intense fire activity occurred during this period, followed by a decrease, with calculated burning temperatures ranging from 295–377°C indicating predominance of surface fires and maybe ground fires [24]. This is in excellent agreement with our results, however our data show continued high wildfire activity in mires during the entire Hettangian (Fig. 5). The causes for the increase in fire activity across the T–J boundary can possibly be linked to two principal factors causing a more wild climate: (1) the establishment of a large sea in a seasonal climate and (2) the contemporaneous CAMP volcanism. It is tempting to select the CAMP volcanism as the main factor as its immediate effect would have been a warmer climate with increased water vapour triggered by extensive CO_2_ and aerosol release. Evaporation from the sea would possibly have accentuated humidity. High water vapour and seasonality likely increased the frequency of thunderstorms and lightning significantly and it is well-established that lightning strikes are the main cause of wildfire ignition [48]. Initially, the floral change to less forested mires may have been a shorter-term effect of SO_2_ pollution associated with CAMP volcanism [7]. The high fire activity possibly helped to sustain more open mire environments, preventing regrowth of arborescent plants. Removal of the canopy may potentially also have made it easier to dry out surface litter in drier months making it more flammable. However, while the regional palynological record indicates early Hettangian recovery of arborescent vegetation, the mire flora remained affected indicating that the environmental changes had a more long-term effect on the sensitive mire ecosystem. The high fire activity and dominance of surface fires during the Hettangian likely indicates a change to at least periodically less waterlogged mires enabling frequent burning of dried surface fuels, maybe promoted by a fluctuating watertable facilitated by a seasonal climate with unevenly distributed rainfall through the year [35]. The T–J boundary was thus a high-stress period that forced the vegetation to adapt to new environmental conditions. Although recovery of the regional terrestrial ecosystem appears to have started already in the early Hettangian [26], recovery of the sensitive mire floras did not commence until around the Hettangian–Sinemurian boundary and decreasing inertinite content through the Lower Jurassic (Fig. 2) suggests that fire activity gradually resumed to considerable lower and possibly more normal levels.
